# Extensive Genetic Diversity of HIV-1 in Incident and Prevalent Infections among Malaysian Blood Donors: Multiple Introductions of HIV-1 Genotypes from Highly Prevalent Countries

**DOI:** 10.1371/journal.pone.0161853

**Published:** 2016-08-30

**Authors:** Wei Zhen Chow, Abdul Hamid Bon, Sheila Keating, Fread Anderios, Hazwan Abdul Halim, Yutaka Takebe, Adeeba Kamarulzaman, Michael P. Busch, Kok Keng Tee

**Affiliations:** 1 Centre of Excellence for Research in AIDS (CERiA), Faculty of Medicine, University of Malaya, Kuala Lumpur, Malaysia; 2 National Blood Centre of Kuala Lumpur (NBCKL), Kuala Lumpur, Malaysia; 3 Blood Systems Research Institute (BSRI), San Francisco, California, United States of America; 4 Department of Laboratory Medicine, University of California, San Francisco (UCSF), California, United States of America; 5 AIDS Research Center, National Institute of Infectious Diseases, Toyama, Shinjuku-ku, Tokyo, Japan; 6 Department of Medical Microbiology, Faculty of Medicine, University of Malaya, Kuala Lumpur, Malaysia; National and Kapodistrian University of Athens, GREECE

## Abstract

Transfusion-transmissible infections including HIV-1 continue to pose major risks for unsafe blood transfusions due to both window phase infections and divergent viruses that may not be detected by donor screening assays. Given the recent emergence of several HIV-1 circulating recombinant forms (CRFs) in high-risk populations in the Southeast Asia region, we investigated the genetic diversity of HIV-1 among the blood donors in Kuala Lumpur, Malaysia. A total of 211 HIV-positive plasma samples detected among 730,188 donations to the National Blood Centre between 2013 and 2014 were provided (90.5% male, median age: 27.0 years old). Recent or long-term infection status at the time of donation was determined using a limiting antigen avidity enzyme immunoassay (LAg-Avidity EIA). HIV-1 *gag-pol* genes were amplified and sequenced from residual plasma for 149 cases followed by genotype determination using phylogenetic and recombination analyses. Transmitted antiretroviral resistance mutations were not observed among the blood donors, among which 22.7% were classified as recent or incident infections. Major circulating HIV-1 genotypes determined by neighbour-joining phylogenetic inference included CRF01_AE at 40.9% (61/149), CRF33_01B at 21.5% (32/149), and subtype B at 10.1% (15/149). Newly-described CRFs including CRF54_01B circulated at 4.0%, CRF74_01B at 2.0%, and CRF53_01B and CRF48_01B at 0.7% each. Interestingly, unique HIV-1 genotypes including African subtype G (8.7%), CRF45_cpx (1.3%), CRF02_AG (0.7%) and CRF07_BC (0.7%) from China were detected for the first time in the country. A cluster of subtype G sequences formed a distinct founder sub-lineage within the African strains. In addition, 8.7% (13/149) of HIV-infected donors had unique recombinant forms (URFs) including CRF01_AE/B' (4.7%), B'/C (2.7%) and B'/G (1.3%) recombinants. Detailed analysis identified similar recombinant structures with shared parental strains among the B'/C and B'/G URFs, some of which were sequenced from recently infected individuals, indicating the possible emergence and on-going spread of foreign clades of CRF candidates among the local population. The findings demonstrate extensive molecular complexity of HIV-1 among the infected blood donors in Malaysia, driven in part by the increased spread of recently described CRFs and multiple introductions of previously unreported genotypes from highly prevalent countries.

## Introduction

In 2014, an estimated 37 million people were living with human immunodeficiency virus-1 (HIV-1) and within the same year, a total of 2 million people were newly diagnosed with HIV-1 [1]. Before the advent of HIV antibody testing in 1985, transfusion-transmissible infections (TTIs) which include HIV-1, hepatitis B virus (HBV) and hepatitis C virus (HCV) were highly prevalent in major parts of the world. A retrospective study conducted on blood component specimens collected during the mid-1980s reported high risk of HIV-1 transfusion from contaminated blood, with approximately 90% of the recipients eventually acquiring HIV-1 infection [2]. Since 1999, the implementation of minipool nucleic acid testing (NAT) has enabled detection of HIV-1 RNA during the highly infectious seronegative window period phase of HIV-1 infection and has greatly reduced the risk of HIV-1 (and HCV) infections due to blood transfusion to 1 in every -12 million units transfused [3–5]. The use of recently developed incidence assays, including that of limiting-antigen avidity enzyme immunoassay (LAg-Avidity EIA) further supplement existing HIV-1 screening and diagnostic assays. Although incidence assays were primarily developed to enable estimation of HIV-1 incidence (defined as the number of new infections during a period of time) in a population [6,7], they also identify recently-acquired (incident) infections in a cross-sectional sample of population. This allows for epidemiological and molecular analyses of the characteristics of recently transmitted HIV-1 infections, enabling more accurate monitoring of the HIV-1 epidemic, thus providing a reliable measure of the impact of preventive measures aimed at reducing HIV-1 transmission, especially in high-risk populations [8].

According to the World Health Organisation (WHO), an estimated 108 million blood donations take place each year [9]. Transfusion of contaminated blood components poses a critical risk of transmitting blood borne infections including HIV, and hepatitis B and C to recipients. In Malaysia, there were an estimated 88,093 people living with HIV-1 by the end of 2014. The early HIV-1 epidemic in Malaysia was largely shaped by unsafe injecting drug use practices, accounting for more than 70% of the total HIV-1 infections in the country. However, the introduction of harm reduction program in 2005 has effectively reduced the HIV-1 prevalence among people who inject drugs (PWIDs) to approximately 16% in 2014 [10,11]. At present, sexual transmission comprise 74% of the total HIV-1 infections in Malaysia [11]. Among the blood donors, a recent analysis estimated HIV-1 incidence rate over a 5-year period (2004 to 2008) at 4–12 infected persons per 100,000 persons years [12].

The HIV-1 epidemic in Malaysia is vastly diverse and involves the co-circulation of three main genotypes which include subtype B, circulating recombinant form 01_AE (CRF01_AE) and CRF33_01B. For example, subtype B (including the Thai variant of subtype B') and CRF01_AE were commonly circulating among men who have sex with men (MSM) and heterosexuals, respectively [13]. Although CRF33_01B co-circulated among various risk populations in the country [14], it has been recently reported to be highly prevalent among the PWIDs [15]. Recently, at least five newly emerging clades of HIV-1 genetic variants circulating at a lower prevalence have been characterised in the country, namely CRF52_01B, CRF53_01B, CRF54_01B, CRF58_01B and CRF74_01B [16–20]. In addition, the continuous genetic recombination involving CRF01_AE and subtype B has also resulted in the emergence of various unique recombinant forms (URFs) displaying distinct mosaic recombination structures in the region [13,15,21].

Despite the recent characterisation of the diverse genetic complexity of HIV-1 among various high-risk populations in Malaysia, the genotypic distribution of HIV-1 among the blood donor population has yet to be investigated. Hence, the aim of this study was to characterise the molecular epidemiological profiles of incident and prevalent HIV-1 infections among a population of blood donors in Kuala Lumpur between 2013 and 2014.

## Materials and Methods

### Ethics statement

The study was approved by the University Malaya Medical Centre (UMMC) Medical Ethics Committee (MEC reference numbers: 733.113, 739.43, and 794.51).

### Study subjects

A total of 730,188 blood donors were screened at the National Blood Centre of Kuala Lumpur (NBCKL) between 2013 and 2014. Plasma specimens were confirmed to be HIV-1 positive by serology testing using the HIV Ag/Ab Combo assay and/or NAT. Briefly, all plasma specimens were screened for TTIs including HIV, HBV and HCV using the ABBOTT PRISM Immunoassay Analyzer (Abbott, Chicago, Illinois, USA). NAT was performed using the Procleix Ultrio Plus assay on the Procleix Tigris system (Grifols Diagnostic Solutions Inc., Emeryville, California, USA) according to the manufacturer’s instructions. During this period, 207 plasma samples were confirmed to be HIV-1 positive based on concordant antibody and RNA positive results and four samples were identified as NAT yield donations based on discordant antibody and RNA results (HIV Ab negative and HIV RNA positive). All residual plasma specimens were stored at -80°C until further processing.

### Viral RNA isolation, amplification and sequencing

HIV-1 RNA was extracted from plasma and reverse-transcribed to cDNA for the amplification and sequencing of the partial *gag-pol* genes (HXB2: 1753–3440). Briefly, total viral nucleic acid was purified from the plasma using NucliSENS easyMAG automated platform (bioMerieux, Durham, North Carolina, USA) and reverse transcribed to cDNA using SuperScript III RNase H^-^ Reverse Transcriptase (Invitrogen, Carlsbad, California, USA) and random hexamers (Applied Biosystems, Foster City, California, USA) according to the manufacturer’s instructions. Nested PCR was performed separately to amplify the partial *gag-pol* gene spanning the *gag* (p24), *protease* (PR) and *reverse transcriptase* (RT) genes using BIO-X-ACT Short DNA Polymerase enzyme (Bioline Reagents Ltd, UK) under the following thermocycling conditions: initial denaturation at 95°C for 5 minutes, followed by 35 cycles of denaturation at 94°C for 30 seconds, annealing at 50°C for 1 minute and elongation at 72°C for 1 minute, and a final elongation step at 72°C for 10 minutes. In order to amplify the *gag*-PR genes, primers 507A (5'-AAG GAA CCC TTT AGA GAC TAT GTA GA-3'; nucleotide positions relative to HXB2: 1657–1682) and 503B (5'-TAT GGA TTT TCA GGC CCA ATT TTT G-3'; HXB2: 2692–2716) were used in the first round of PCR, followed by primers 508A (5'-GTA AAA AAT TGG ATG ACA GAA ACC TTG-3'; HXB2: 1726–1752) and 504B (5'-ACT TTT GGG CCA TCC ATT CC-3'; HXB2: 2611–2592) in the second round of PCR as described previously [15]. In a separate reaction, the RT gene was amplified using primers 325A (5'-GGA AAC CAA AAA TGA TAG GGG GAA TTG GAG G-3'; HXB2: 2377–2407) and 326B (5'-CTG TAC TTC TGC TAC TAA GTC TTT TGA TGG G-3'; HXB2: 3539–3509) in the first round of PCR and primers 327A (5'-GTG GAA AAA AGG CTA TAG GTA CAG-3'; HXB2: 2452–2475) and 328B (5'-CTG CCA ACT CTA ATT CTG CTT C-3'; HXB2: 3462–3441) were used in the second round of PCR. Population sequencing of the purified PCR products was performed using an ABI PRISM 3730XL DNA Analyzer (Applied Biosystems, USA).

### Phylogenetic and recombination analysis

Partial *gag-pol* nucleotide sequences of approximately 1.6kb in sequence length (HXB2: 1753–3440) were assembled using DNASIS Max (Hitachi, Japan) and aligned with relevant global HIV-1 reference subtypes (B, C, D, and G) and CRFs (CRF01_AE, CRF33_01B, CRF34_01B, CRF48_01B, CRF53_01B, CRF54_01B, CRF58_01B and CRF74_01B from Southeast Asia; CRF07_BC, CRF08_BC, CRF57_BC, CRF61_BC, CRF62_BC and CRF64_BC from China; CRF60_BC from Italy; and CRF02_AG and CRF45_cpx from Africa) downloaded from the Los Alamos HIV database (http://www.hiv.lanl.gov/). Sequences were manually adjusted using BioEdit 7.0 with reference to the HIV Sequence Compendium 2014 (http://www.hiv.lanl.gov/) to ensure an accurate codon alignment. Phylogenetic trees were constructed by the neighbour-joining method based on the Kimura two-parameter model with a transition-transversion ratio of 2.0 implemented in MEGA 5.05 [22]. The reliability of the branching orders were analysed by bootstrap analysis of 1000 replicates. Nucleotide sequences obtained in the study were analysed for the presence of unique recombinant structures using the Recombinant Identification Program (RIP) available at the Los Alamos HIV database. Bootscanning and informative-site analyses [23] were performed on recombinant genotypes using SimPlot version 3.5.1 [24]. In order to confirm the putative parental origin of each recombinant segment, sub-region neighbour-joining trees were constructed in MEGA. The newly-generated partial *gag-pol* nucleotide sequences were submitted in the GenBank under the following accession numbers: KU535900 to KU535913 and KU535918 to KU536052.

### Identification of incident and prevalent HIV-1 infections using a limiting-antigen avidity assay

The LAg-Avidity EIA was used to discriminate incident and prevalent HIV-1 infections among a subset of the blood donor population with available index donation plasma specimens (n = 179). The assay was performed using the HIV-1 LAg-Avidity EIA kit developed by the US Centers for Disease Control and Prevention (US CDC) (Sedia Biosciences Corporation, Oregon, USA) which incorporates the use of a single-well avidity assay with limiting antigen comprising of a newly-developed recombinant protein (rIDR-M) representing major variants of gp41 immunodominant regions in the HIV-1 group M [7]. Briefly, 100 μL of the diluted controls, calibrators and plasma (1:101) were incubated for an hour at 37°C. A 200 μL of Dissociation Buffer was added and the plate was incubated for 15 minutes at 37°C. The conjugate working solution was prepared by diluting the goat anti-human IgG-HRP (horseradish peroxidase) conjugate reagent (1:1001) in phosphate buffer saline (PBS). Next, 100 μL of conjugate working solution was added and the plate was incubated for 30 minutes at 37°C. A 100 μL of 3,3',5,5'-tetramethylbenzidine substrate was added followed by incubation at 25°C for 15 minutes. The plate was washed four times with PBS after each incubation step prior to the addition of reagents. Finally, 100 μL of Stop Solution was added and the optical density (OD) of each reaction was read at 450nm using a spectrophotometer. The measured OD was normalized (ODn) against the median OD of the calibrator. All specimens were tested in singlet while the controls and calibrators were tested in triplicates. A long-term infection was identified based on an ODn >2.0 and further confirmatory tests were conducted in triplicates if the ODn ≤2.0, in which a median ODn ≤1.5 indicates a recent infection [6].

## Results

The study population comprised of 211 adults who donated blood at the NBCKL between 2013 and 2014. Prior donation status was available for 64.5% (n = 136) of the study population: 41.7% (n = 88) were first-time donors, 21.8% (n = 46) were regular repeat donors and 0.9% (n = 2) were lapsed donors (>1 year between previous seronegative and index seropositive donations). The study population comprised mainly of males (n = 191, 90.5%). with a median age of 27.0 years (interquartile range, IQR: 23.0–32.0 years) and of the Malay ethnicity (n = 92, 43.6%), followed by the Chinese (n = 25, 11.8%), Indians (n = 16, 7.6%) and others from East Malaysia (Sabah and Sarawak) (n = 16, 7.6%). Around 5–8% of the study population comprised of men who had sex with men (MSM) (n = 11) and heterosexuals (n = 17). Information regarding the ethnicity and route of HIV-1 transmission was unavailable for 29.3% (n = 62) and 89.1% (n = 188), respectively of the study population. Overall, the rates of co-infections was higher for syphilis at 9.0% (n = 19), compared to HBV (n = 3, 1.4%) or HCV (n = 1, 0.5%).

**Fig 1:**
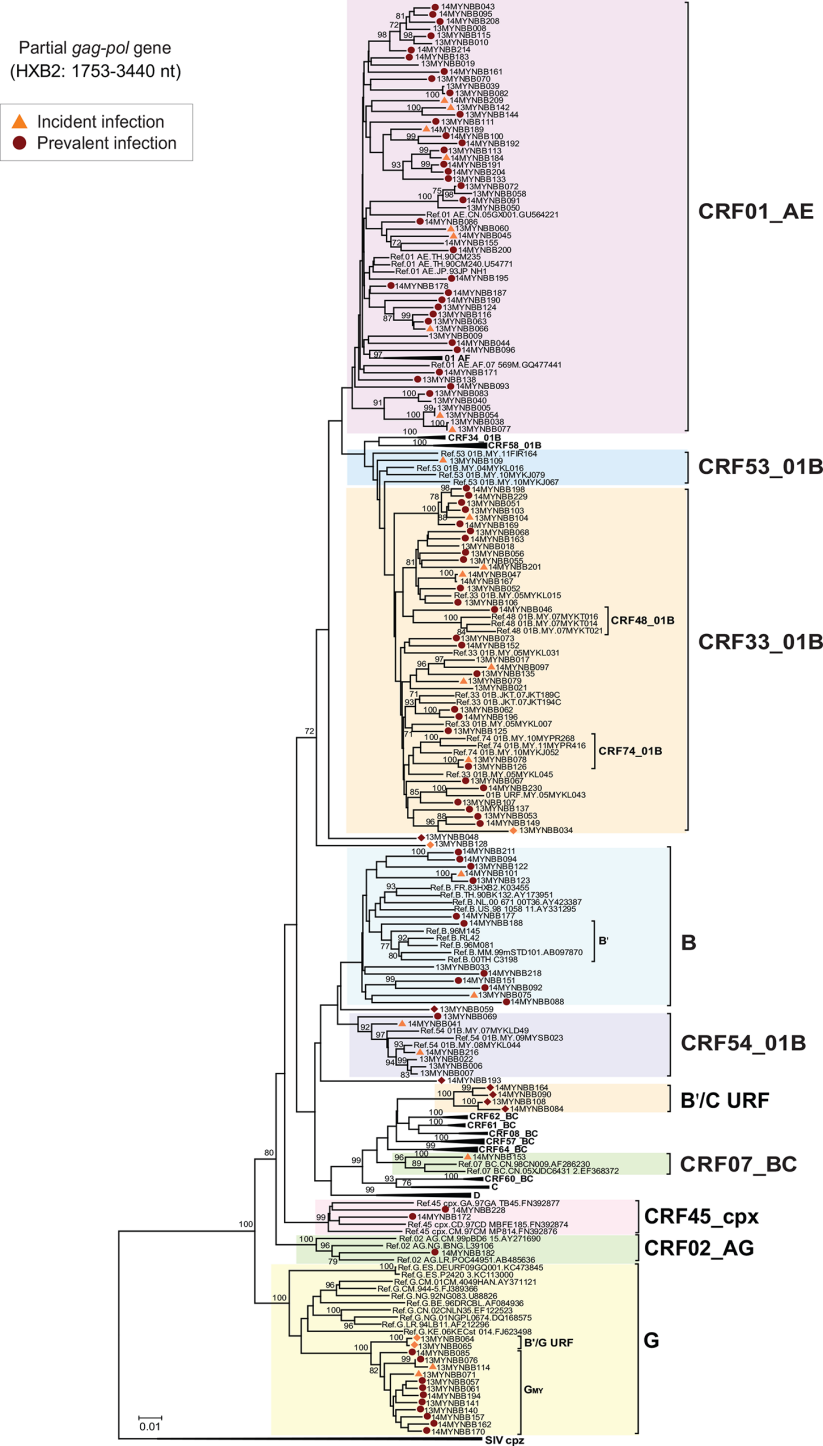
Phylogenetic reconstruction of 136 partial *gag-pol* gene sequences of 1.6kb amplified among the blood donors in Kuala Lumpur, Malaysia between 2013 and 2014. HIV-1 incidence was estimated using a limiting antigen avidity enzyme immunoassay (LAg-Avidity EIA) to identify recent (incident) and long-standing (prevalent) infections as indicated where available. Neighbour-joining tree was constructed in MEGA 5.05 [22] using Kimura 2-parameter method of nucleotide substitutions and the reliability of the branching nodes were assessed by bootstrap analysis of 1000 replicates. Eleven partial *gag*-PR (834bp) and two RT gene sequences (966bp) were genotyped separately using similar methods and their prevalence was reported in this study (figures not shown for clarity). Relevant HIV-1 reference genotypes in Southeast Asia include subtype B, CRF01_AE, CRF33_01B, CRF34_01B, CRF48_01B, CRF52_01B, CRF53_01B, CRF54_01B, CRF58_01B and CRF74_01B. Reference sequences of other genotypes prevalent in China (CRF07_BC, CRF08_BC and other recently-described B'/C CRFs) and Africa (subtype G, CRF02_AG and CRF45_cpx) were also included in the analysis. The reference sequences were labelled in the following order: genotype, country of origin, isolate name and GenBank accession number. A well-supported cluster of Malaysian subtype G strains was also highlighted as G_MY_ within the subtype G clade of African reference strains. All 12 unique recombinant forms were denoted by closed diamonds and labelled according to incident or prevalent infection status. Clusters of novel B'/C recombinants (strains 13MYNBB108, 14MYNBB084, 14MYNBB090 and 14MYNBB164) and B'/G recombinants (13MYNBB064 and 13MYNBB065) were highlighted in the tree. Simian immunodeficiency virus (SIVcpz) reference strains were included as outgroup. Bootstrap values of greater than 70% were indicated on the branch nodes. The scale bar represents 1% genetic distance (0.01 substitutions per site).

Out of 211 samples processed, a total of 149 (70.6%) partial *gag-pol* gene sequences were successfully amplified and 91.3% (n = 136/149) of these sequences were assembled to generate up to 1.6kb partial *gag-pol* sequences (HXB2: 1753–3440). On the other hand, 11 partial *gag*-PR (HXB2: 1753–2591, 834bp) and 2 RT gene (HXB2: 2476–3440, 966bp) sequences were also amplified. Transmitted antiretroviral drug resistance mutation was not detected in the population, similar to previous drug resistance surveillance studies reported among antiretroviral-naïve patients in the country [13,25]. Based on the neighbour-joining phylogenetic tree analysis, HIV-1 genotypes mainly co-circulating in the region including CRF01_AE was detected among 40.9% (n = 61) of the blood donors, followed by CRF33_01B (n = 32, 21.5%) and subtype B (n = 15, 10.1%, including the subtype B variant of Thai origin, B') (**Fig 1**). In addition, at least four newly-emerging CRFs previously reported in various risk populations in the country were detected at a low prevalence in the population, namely CRF54_01B (n = 6, 4.0%), CRF74_01B (n = 3, 2.0%), CRF48_01B (n = 1, 0.7%) and CRF53_01B (n = 1, 0.7%). On the other hand, CRF15_01B, CRF34_01B, CRF51_01B, CF52_01B and CRF58_01B that have been reported in the Southeast Asian region were not detected. In addition to the local HIV-1 genotypes, interestingly, we also identified the co-circulation of four imported genotypes for the first time in the country, comprising of subtype G (n = 13, 8.7%), CRF45_cpx (n = 2, 1.3%) and CRF02_AG (n = 1, 0.7%) from Africa, and CRF07_BC (n = 1, 0.7%) from China (**Fig 1**).

**Fig 2:**
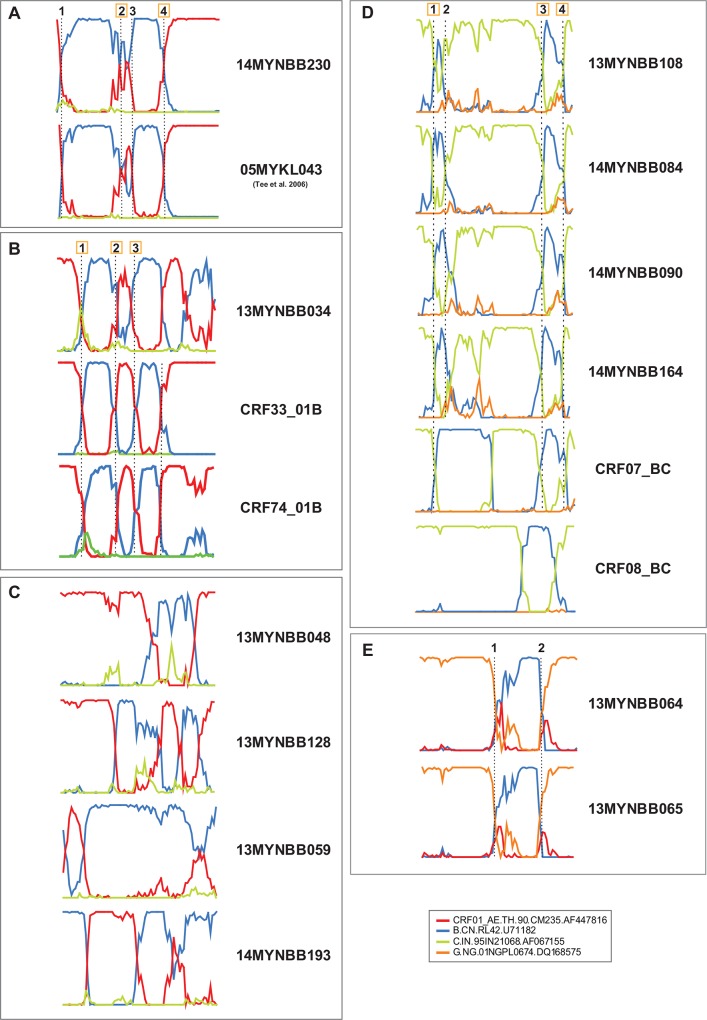
Bootscan and informative sites analyses of the 1.6kb partial *gag-pol* gene sequences of various HIV-1 unique recombinant forms (URFs) characterised among the blood donors in Kuala Lumpur. **A,** Bootscan plot of strain 14MYNBB230 which shares all four recombination breakpoints with a previously reported URF, 05MYKL043 (GenBank accession number: DQ366666) [14]. Both strains also share two identical breakpoints with CRF33_01B, as denoted by square textboxes. **B,** Bootscan plot of strain 13MYNBB034 which displays a total of six recombinant segments involving CRF01_AE and subtype B' (of Thai origin), whereby three breakpoints were identical to that of CRF33_01B and CRF74_01B (which shares four breakpoints with CRF33_01B). Strain 13MYNBB034 may be another newly-characterised genetic variant of CRF33_01B which differed structurally from CRF74_01B. **C,** Bootscan plots of four CRF01_AE/B' URFs (13MYNBB048, 13MYNBB128, 13MYNBB059 and 14MYNBB193) displaying distinct mosaic recombination structures and breakpoints, which have yet to be reported in the country. **D,** Bootscan plots of subtype B'/C recombinants displaying all identical recombination structures and breakpoints amongst each other which potentially represent a novel CRF candidate in Kuala Lumpur. All four strains which are epidemiologically-unlinked (13MYNBB108, 14MYNBB084, 14MYNBB090 and 14MYNBB164) also shared three breakpoints (as denoted by square textboxes) with CRF07_BC which is prevalent in China [32]. These isolates, however were genetically and structurally distinct from CRF08_BC from China. **E,** Bootscan plots of subtype B'/G recombinants (13MYNBB064 and 13MYNBB065) displaying all identical recombination structures and breakpoints amongst each other. Putative HIV-1 parental reference genotypes were selected by similarity plot, which included 90THCM235 (CRF01_AE), CNRL42 (subtype B' of Thai origin), 95IN21068 (subtype C) and 01NGPL0674 (subtype G). All breakpoints were labelled numerically and identical breakpoints were highlighted with dotted lines. Bootscan was performed in SimPlot version 3.5.1 [24] using a window size of 200 nucleotides moving along the alignment in increments of 20 nucleotides.

Using RIP to screen all partial *gag-pol* sequences for evidence of recombination, followed by SimPlot, bootscan and informative sites analyses to assign the putative parental reference strains (CRF01_AE.90THCM235 and subtype B.CNRL42) and estimate the recombination breakpoints, we first identified a CRF01_AE/B' URF (14MYNBB230) that clustered with a previously reported URF (05MYKL043, GenBank accession number: DQ366666) [14] within the CRF33_01B clade with a strong bootstrap support (**Fig 1**). Both strains shared identical mosaic recombination structures and breakpoints, comprised of two subtype B' fragments of 435bp and 379bp at HXB2 positions 1940–2374 nt and 2462–2840 nt and three CRF01_AE segments of 151bp, 25bp and 565bp at positions 1753–1903 nt, 2417–2441 nt and 2876–3440 nt, respectively (**Fig 2A**). In addition, these strains also shared identical recombination breakpoints (second and fourth) with CRF33_01B in the partial *gag-pol* genes, at HXB2 positions 2375–2416 nt and 2841–2875 nt, respectively. Next, also within the CRF33_01B clade, RIP analysis revealed that strain 13MYNBB034 showed unique CRF01_AE/B' recombination structure distinct from other CRF33_01B strains. Three subtype B' fragments of 311bp, 289bp and 280bp at positions 2064–2374 nt, 2552–2840 nt and 3059–3338 nt and three CRF01_AE fragments of 300bp, 105bp and 82bp at positions 1753–2052 nt, 2916–3020 nt and 3356–3437 nt, respectively were identified in 13MYNBB034 (**Fig 2B**). Detailed recombination analysis revealed that the first three breakpoints characterised in 13MYNBB034 at positions 2053–2063 nt, 2375–2422 nt and 2538–2551 nt were identical to that of CRF33_01B.

Given the fact that the partial *gag-pol* genes in CRF74_01B had been previously characterised to comprise of a total of six fragments (three subtype B' and three CRF01_AE fragments), of which the first four breakpoints were identical to that of CRF33_01B [15], we performed further comparative recombination analysis of the breakpoints between 13MYNBB034 and CRF74_01B reference strains. Our analysis revealed the third subtype B' fragment in 13MYNBB034 (HXB2: 3059–3338 nt) was relatively longer by 158bp compared to that of CRF74_01B (HXB2: 3161–3283 nt, 123bp). Additional phylogeny inference of the partial *gag-pol* sequences of 13MYNBB034 and relevant reference strains indicated that 13MYNBB034 was located outside the CRF33_01B and CRF74_01B clades (**S1 Fig**). Altogether, our analyses suggest that 13MYNBB034 could be another genetic variant of CRF33_01B, which was genetically-distinct from other CRF33_01B-related CRFs and URFs reported so far in the country.

Four other strains (13MYNBB048, 13MYNBB128, 13MYNBB059 and 14MYNBB193) did not cluster with any known HIV-1 reference genotypes in the neighbour-joining tree (**Fig 1**). Located outside the CRF33_01B clade, strain 13MYNBB048 was comprised of a subtype B' fragment of 381bp at position 2727–3107 nt and two CRF01_AE fragments of 938bp and 298bp at positions 1753–2690 nt and 3143–3440 nt, respectively (**Fig 2C**). Next, in 13MYNBB128, two short subtype B' fragments of equal length (322bp) were characterised at positions 2426–2747 nt and 2939–3260 nt, followed by three CRF01_AE segments of 543bp, 142bp and 157bp at positions 1753–2295 nt, 2783–2924 nt and 3284–3440 nt, respectively. In strain 13MYNBB059 located outside the subtype B clade, a longer subtype B' (1377bp) and short CRF01_AE fragments (148bp) were identified at positions 2064–3440 nt and 1839–1986 nt, respectively. Finally, located outside the B'/C CRFs and subtype D clades was strain 14MYNBB193 which was comprised of three subtype B' fragments of 228bp, 346bp and 369bp at positions 1770–1997 nt, 2552–2897 nt, 3032–3400 nt and two CRF01_AE fragments of 522bp and 81bp at positions 2016–2537 nt and 2916–2996 nt, respectively (**Fig 2C**).

**Fig 3:**
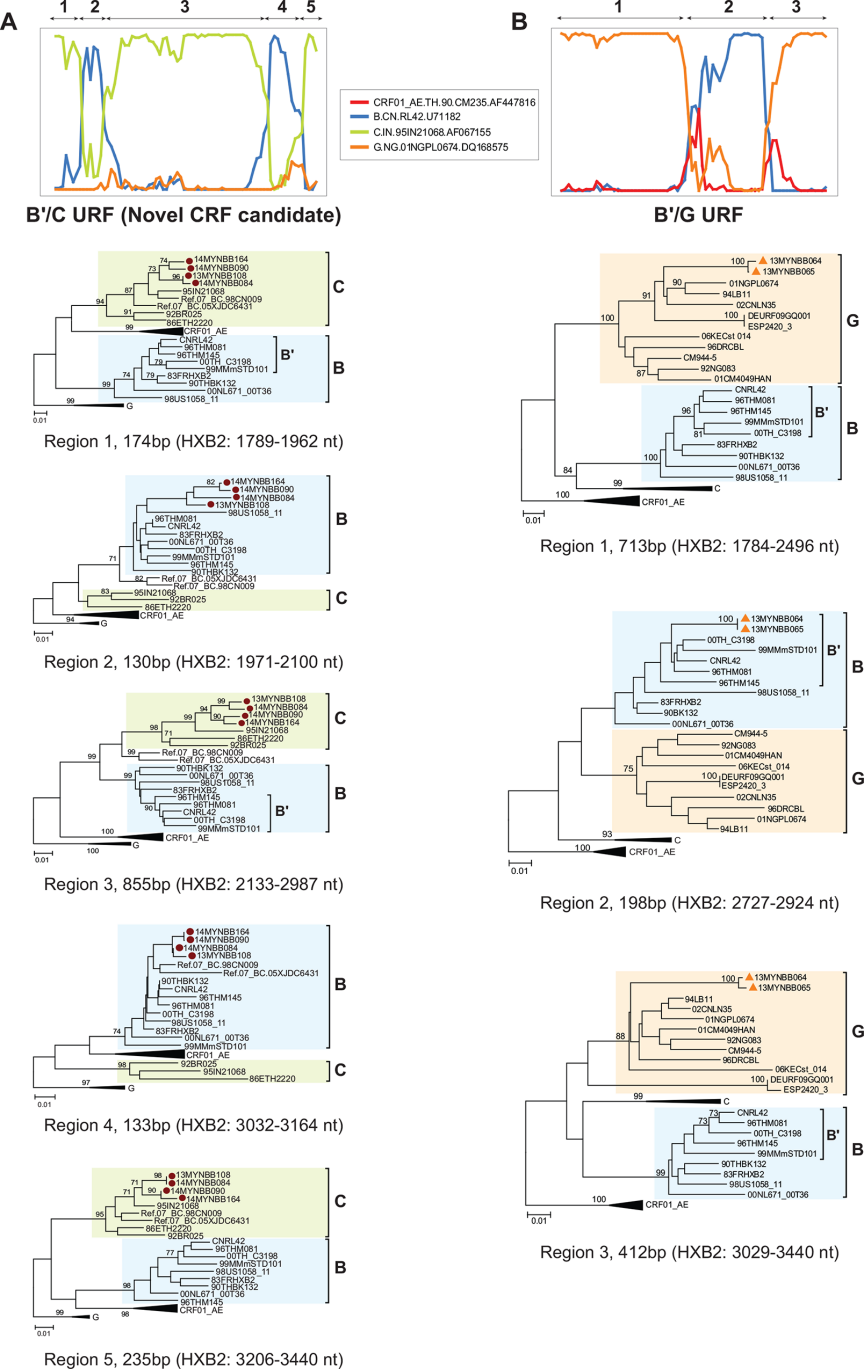
**Sub-region neighbour joining tree analyses of the 1.6kb partial *gag-pol* genes sequenced in two clusters of (A) subtype B'/C and (B) B'/G recombinants characterised in the population.** Based on the informative sites analyses, recombination breakpoints were estimated for each strain and the partial *gag-pol* sequences (HXB2:1753–3440) were then sub-divided into different regions for phylogenetic reconstruction. Putative HIV-1 parental reference genotypes used in bootscan were 90THCM235 (CRF01_AE), CNRL42 (subtype B' of Thai origin), 95IN21068 (subtype C) and 01NGPL0674 (subtype G). Incident or prevalent HIV-1 infections for each strain, as determined using a limiting antigen avidity enzyme immunoassay (LAg-Avidity EIA) were identified by orange triangles or red circles, respectively. Bootstrap values of greater than 70% were indicated on the branch nodes. The scale bar represents 1% genetic distance (0.01 substitutions per site).

Interestingly, four strains (13MYNBB108, 14MYNBB084, 14MYNBB090 and 14MYNBB164) formed a monophyletic cluster (with bootstrap support of 100%) which appeared to be distinct lineages from the seven previously characterised B'/C CRFs from China including CRF07_BC, CRF08_BC, CRF57_BC, CRF61_BC, CRF62_BC and CRF64_BC, and also CRF60_BC from Italy (**Fig 1**). Detailed recombination analysis of the partial *gag-pol* genes using the putative parental reference strains (subtype B.CNRL42 and subtype C.95IN21068) and a subtype G reference strain as an outgroup (01NGPL0674), documented identical mosaic recombination structures and breakpoints shared between the four epidemiologically-unlinked strains, suggestive of a novel CRF candidate (**Fig 2D**). A total of two subtype B' fragments of 130bp and 133bp at positions 1971–2100 nt and 3032–3164 nt and three subtype C fragments of 174bp, 855bp and 235bp were characterised at positions 1789–1962 nt, 2133–2987 nt and 3206–3440 nt, respectively, in all four strains and the subtype origin of each fragment was confirmed using sub-region neighbour joining trees (**Fig 3A**). In order to determine whether these strains share an evolutionary relationship with CRF07_BC or CRF08_BC, we re-analysed the recombination breakpoints in the partial *gag-pol* genes of both reference strains. Interestingly, we observed that the B'/C URF indeed shared at least three breakpoints with CRF07_BC at positions 1963–1970 (±20) nt, 2988–3001 (±10) nt and 3165–3205 nt, as indicated in **Fig 2D**. Moreover, sub-region phylogenetic inference of regions 1, 4 and 5 in the B'/C URF showed close clustering with the corresponding fragments in CRF07_BC (**Figs 2D and 3A**) in the partial *gag-pol* gene. Altogether, the results indicated a plausible evolutionary relationship between the newly-sequenced CRF candidate strains and CRF07_BC which necessitates further confirmation through the sequencing and recombination analysis of the near full length genomes.

We also identified a cluster (with bootstrap support of 80%) of subtype G among the Malaysian blood donors (subtype G_MY_) which formed a distinct sub-lineage from other subtype G reference strains of African origin (subtype G_AF_), suggesting a potential founder effect among the strains. To further characterize the spread of subtype G_MY_ in the population, we performed phylogenetic reconstruction and estimated the intra-group genetic distances of the partial *gag-pol* sequences of the subtype G_MY_ strains and a total of 75 subtype G_AF_ reference strains downloaded from GenBank (**S2 Fig**). Intra-group genetic distances for subtype G_MY_ and subtype G_AF_ sequences were 2.6 ± 0.2% and 7.3 ± 0.3% nucleotide substitutions per site, which possibly indicated a more recent spread of subtype G in the Malaysian blood donors. Located outside the subtype G_MY_ cluster, a well-supported cluster (100% bootstrap support) of strains 13MYNBB064 and 13MYNBB065 was observed. Detailed recombination analysis using subtype B.CNRL42 and subtype G.01NGPL0674 as the putative parental reference strains identified identical recombination structures and breakpoints between both strains, characterised as B'/G recombinants (**Fig 2E**). Partial *gag-pol* genes of these strains were comprised of a 198bp subtype B' fragment at position 2727–2924 nt and two subtype G fragments of 713bp and 412bp at HXB2 positions 1784–2496 nt and 3029–3440 nt, respectively, as confirmed by sub-region tree analysis (**Fig 3B**).

**Fig 4:**
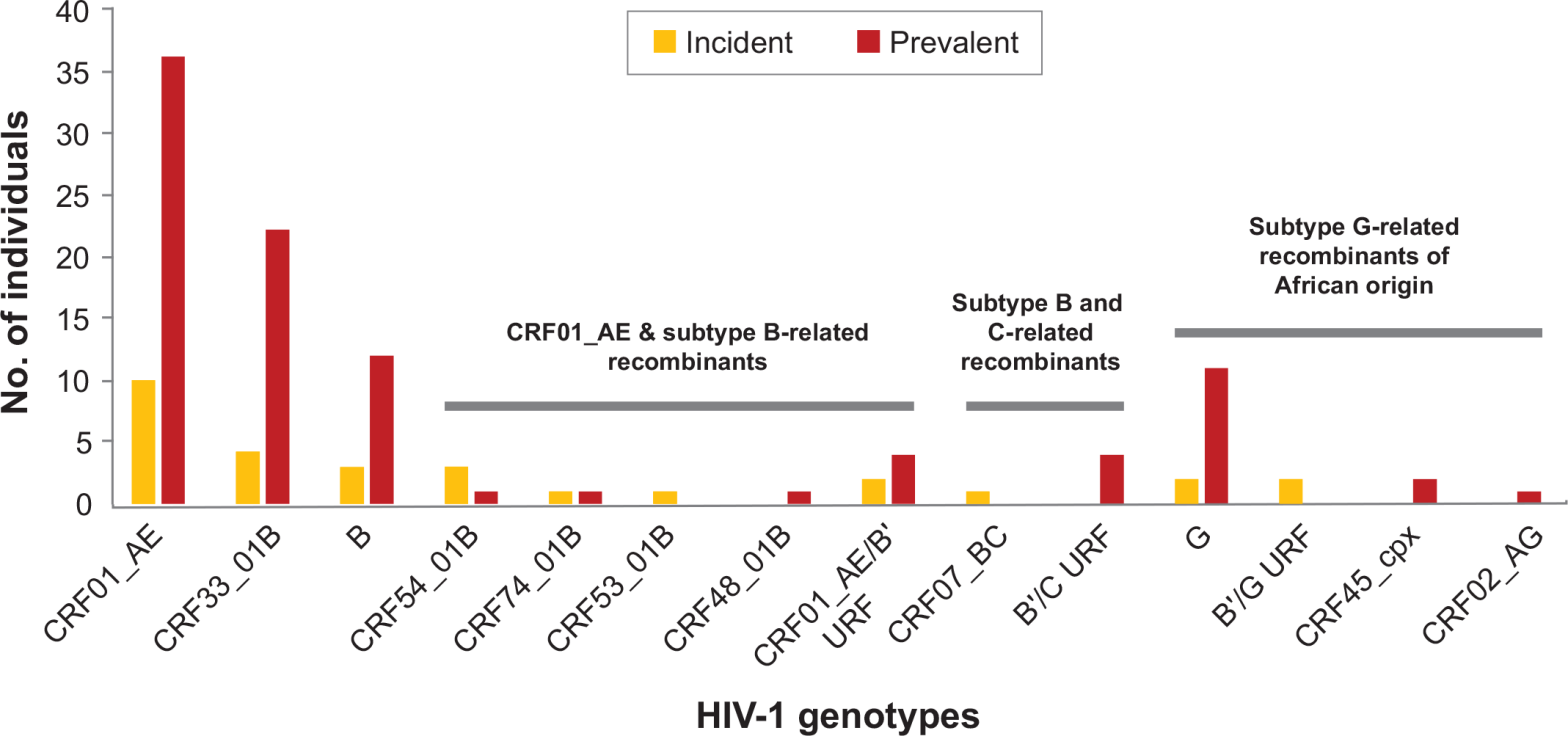
HIV-1 genotype distribution of incident and prevalent infections among 127 blood donors in Kuala Lumpur. A limiting-antigen avidity enzyme immunoassay (LAg-Avidity EIA) was used to distinguish incident from prevalent HIV-1 infections. Out of 179 samples available for incidence assay testing, 70.9% (n = 127) were successfully genotyped and comprised of 29 (22.8%) incident HIV-1 infections.

A total of 179 (84.8%) plasma samples were available for LAg incidence assay testing to identify incident and prevalent HIV-1 infections in the study population, of which 22.7% (n = 48) were classified as incident infections (probable antibody seroconversion within the previous 3–4 months). Among the samples with LAg results, 70.9% (n = 127/179) were successfully genotyped and 22.8% (n = 29/127) were incident HIV-1 infections. The HIV-1 genotype distribution among both incident and prevalent HIV-1 infections were diverse (**Fig 4**). In particular, at least two subtypes, six CRFs and various distinct URFs were detected in recently-infected blood donors. A total of 37.9% (n = 11/29) of incident infections were comprised of the major circulating genotypes, CRF01_AE, followed by CRF33_01B (17.2%, n = 5) and subtype B (6.9%, n = 2). Other genotypes which were also detected at a lower prevalence in incident infections include CRF54_01B (6.9%, n = 2), subtype G (6.9%, n = 2), and CRF74_01B, CRF53_01B and CRF07_BC at 3.4% (n = 1) each. Moreover, CRF01_AE/B' and B'/G URFs were each circulating in 6.9% (n = 2) of the recently-infected blood donors (**Fig 4**).

## Discussion

We hereby report a molecular epidemiological surveillance analysis of HIV-1 strains among a subset of HIV-1 infected blood donors recruited in Kuala Lumpur between 2013 and 2014, using a set of phylogenetic and recombination analysis methods. Besides identifying the co-circulation of major local HIV-1 genotypes and other newly-emerging CRFs in the population, we also reported, for the first time, the significant importation of various genetic lineages from countries where HIV-1 infection is highly prevalent. The use of a recently developed incidence assay (LAg-Avidity EIA) also enabled the discrimination of recently acquired from established infections in the HIV-positive donor population, for correlation with HIV-1 genetic diversity.

Based on phylogenetic reconstruction of the partial *gag-pol* genes, we identified three main HIV-1 genotypes commonly circulating in various risk populations, including CRF01_AE, subtype B (including subtype B') and CRF33_01B [13–15] which attributed to around 73% of the total HIV-1 infections among the blood donors. We observed a higher prevalence of the Western subtype B (around 10%) compared to subtype B', which was reportedly circulating predominantly among the MSM populations in the country and elsewhere in the region [13,26]. Of note, we also observed an increased frequency of CRF54_01B (around 4%) which was previously described at a lower frequency among various risk populations [13,18], indicating the establishment of CRF54_01B in the general population and its possible expansion as one of the major recombinant genotypes in the country. On the other hand, at least three previously identified genetic clades of CRFs which share structural and evolutionary relationship with CRF33_01B, including CRF48_01B, CRF53_01B and CRF74_01B, continue to circulate at a lower prevalence (<2%), similar to earlier reports [19,20,27]. In fact, the co-circulation of CRF01_AE, CRF33_01B, and subtype B' continues to spur the increasingly complex genetic diversity of HIV-1 in Kuala Lumpur [15,28], as evident in the newly characterised CRF01_AE/B' recombinants (13MYNBB034 and 14MYNBB230) which share at least two identical recombination breakpoints with CRF33_01B in the partial *gag-pol* genes, and other unique CRF01_AE/B' recombinants with distinct recombination structures (13MYNBB048, 13MYNBB128, 13MYNBB059 and 14MYNBB193). Besides its predominance in the PWIDs [15], our findings also indicate the epidemiological significance of CRF33_01B which is most likely to remain as the main co-circulating recombinant genotype transmitted among various risk populations in the country.

In East Asia, the early HIV-1 epidemic in China involved the co-circulation of subtype B' and subtype C (of Indian origin), particularly in the Dehong prefecture of Yunnan province (bordering the “Golden Triangle”), as a result of intense drug trafficking activities in the early 1990s [29]. As a result, various subtype B'/C recombinants emerged among PWIDs thereafter [30], including the two predominant circulating genotypes believed to be originating from Western Yunnan [31], CRF07_BC [32] and CRF08_BC [33]. In the present study, we reported the first identification of CRF07_BC in a recently-infected blood donor, probably as a result of sporadic introduction of CRF07_BC lineage into the country. However, it is to our surprise that a cluster of B'/C recombinants (13MYNBB108, 14MYNBB084, 14MYNBB090 and 14MYNBB164) that shared identical mosaic recombination structures was identified, in which three of the estimated breakpoints in the *gag-pol* genes were identical to that of CRF07_BC, suggestive of a close evolutionary relationship between the potential novel CRF candidate and CRF07_BC. Due to the very low frequency of CRF07_BC and subtype C in the country (previously estimated around 2% in the general population [13]), it remains unclear if the B'/C unique recombinants were generated through inter-subtype recombination between the local subtype B', C and/or CRF07_BC strains circulating in the country. Therefore, there is a likelihood that the B'/C URFs were probably imported from areas where the putative parental strains were endemic, such as countries in East Asia. Interestingly, recent reports have shown a significant increase in B'/C inter-subtype recombinants [34,35], some of which were classified as CRF [36–39], emerging in various parts of China. Hence, the detection of B'/C unique recombinants in Malaysia is probably due to cross-border transmission of these recombinants, which are widespread in China, into neighbouring countries in Southeast Asia. However, when compared with all published B'/C recombinant sequences from China (data not shown), the newly identified B'/C recombinants did not share similar recombination structures with the Chinese B'/C URFs, indicating that the Malaysian strains may represent a yet to be identified recombinant lineage circulating in the region. More detailed analyses are nonetheless necessary to better understand the recombinant lineage. The availability of the full length genomes will allow the classification of the Malaysian B'/C URFs as a novel CRF lineage [40], and subsequently enable a more comprehensive phylodynamic mapping to identify the origin and transmission dynamics of the lineage within and between countries in the region [20].

The extensive genetic diversity of HIV-1 among the blood donors in Kuala Lumpur was further documented by the detection of subtype G and other CRFs including CRF02_AG [41] and CRF45_cpx [42], which circulate predominantly in West and Central Africa [43]. Altogether, these genotypes comprised ~10% of the total HIV-1 infections in the study population. HIV-1 subtype G originated in Central Africa around the late 1960s, before it expanded to other regions within Africa [44] and later globally [45–47], with limited circulation in East Asia [48,49]. On the other hand, CRF45_cpx remained endemic in the continent and was rarely reported outside Africa [50]. Previous epidemiological surveillance conducted in various risk populations in Kuala Lumpur [13,15] did not detect the circulation of subtype G and CRF45_cpx, hence our findings herein provide the first genetic evidence of subtype G and CRF45_cpx introduction into the Malaysian population. The frequency and apparent founder effect shown by the subtype G_MY_ sub-lineage within the African strains further suggest the expansion and local spread of subtype G in the country. In addition, we also characterised a cluster of two subtype B'/G recombinants (strains 13MYNBB064 and 13MYNBB065) among incident infections which may indicate an on-going inter-subtype recombination between local subtypes B' and G at a low rate in the infected blood donor population.

Molecular epidemiological studies provide an effective strategy to detect the major circulating HIV-1 strains in infected blood donors, as well as to detect newly-emerging or unique viral strains [51]. Moreover, in populations where access to antiretroviral therapy has been scaled up, continuous surveillance studies are important to identify transmitted drug resistance mutations, which are essential to guide the early implementation of antiretroviral therapy in HIV-infected blood donors [52,53]. Of note, the molecular epidemiological findings observed in this study may have important implications on blood donation screening. The increasingly diverse HIV-1 genotypes in the blood donor population, of which around 23% were recently-infected may pose a challenge in the early detection and diagnosis of HIV-1 infection using commercially-available assays. For instance, routine HIV antigen/antibody assays were mainly developed and evaluated based on the subtype B strains. As a result, the analytical sensitivity of these assays may vary in detecting a broad array of non-B HIV-1 genotypes, leading to potential false negative interpretation [54,55]. The LAg-Avidity EIA had been shown to be efficient in distinguishing recent from long-term infections involving various non-B, group M genotypes [7,56] through the incorporation of a multi-subtype gp41 recombinant protein in the assay, hence increasing the sensitivity of early HIV-1 detection. However, in view of recent reports of misclassification by LAg-Avidity EIA in populations infected with B and non-B subtypes [57,58], further studies involving longitudinal populations and diverse HIV-1 genotypes are necessary.

In conclusion, our results demonstrate extensive molecular complexity of HIV-1 among both recently and long-term infected blood donors, with a total of eleven subtypes/CRFs and various distinct URFs detected in Kuala Lumpur, Malaysia. This was driven in part by the increased spread of recently described CRFs, but also by multiple introductions of previously unreported genotypes from high prevalence countries. Altogether, the genetic data generated herein may be used to inform future assessment and development of a more sensitive blood screening and supplemental assays targeted at major circulating strains in the general population. Continuous molecular surveillance of HIV-1 among blood donors is thus imperative in order to ensure the safety of blood transfusion in Malaysia and worldwide.

## Supporting Information

S1 Fig Neighbour-joining tree of partial *gag-pol* sequences of 13MYNBB034 and other CRFs including CRF33_01B and CRF74_01B, previously characterised in Kuala Lumpur. Phylogenetic tree was constructed in MEGA 5.05 using Kimura 2-parameter method of nucleotide substitutions and the reliability of the branching nodes were assessed by bootstrap analysis of 1000 replicates. The reference sequences were labelled in the following order: country of origin, year, isolate name and GenBank accession number. Abbreviations were used to indicate the country of origin. Bootstrap values of greater than 70% were indicated on the branch nodes. The scale bar represents 1% genetic distance (0.01 substitutions per site).
(PDF)

S2 Fig Phylogenetic reconstruction of partial *gag-pol* sequences of subtype G_MY_ clade (n = 12) and publicly available subtype G_AF_ reference sequences (n = 75) downloaded from GenBank. Neighbour-joining tree was constructed in MEGA 5.05 using Kimura 2-parameter method of nucleotide substitutions and the reliability of the branching nodes were assessed by bootstrap analysis of 1000 replicates. The reference sequences were labelled in the following order: genotype, country of origin, isolate name and GenBank accession number. Abbreviations used include MY, Malaysia and AF, Africa. Subtype B reference sequences were used as outgroup. Bootstrap values of greater than 70% were indicated on the branch nodes. The scale bar represents 1% genetic distance (0.01 substitutions per site).
(PDF)
