# Supplementary figures and images for: Extensive Genetic Diversity of HIV-1 in Incident and Prevalent Infections among Malaysian Blood Donors: Multiple Introductions of HIV-1 Genotypes from Highly Prevalent Countries
Source: PLoS One. 2016 Aug 30;11(8):e0161853. doi: 10.1371/journal.pone.0161853 (PMC5004849; doi:10.1371/journal.pone.0161853)

Partial *gag-pol* gene  
(HXB2: 1753-3440 nt)

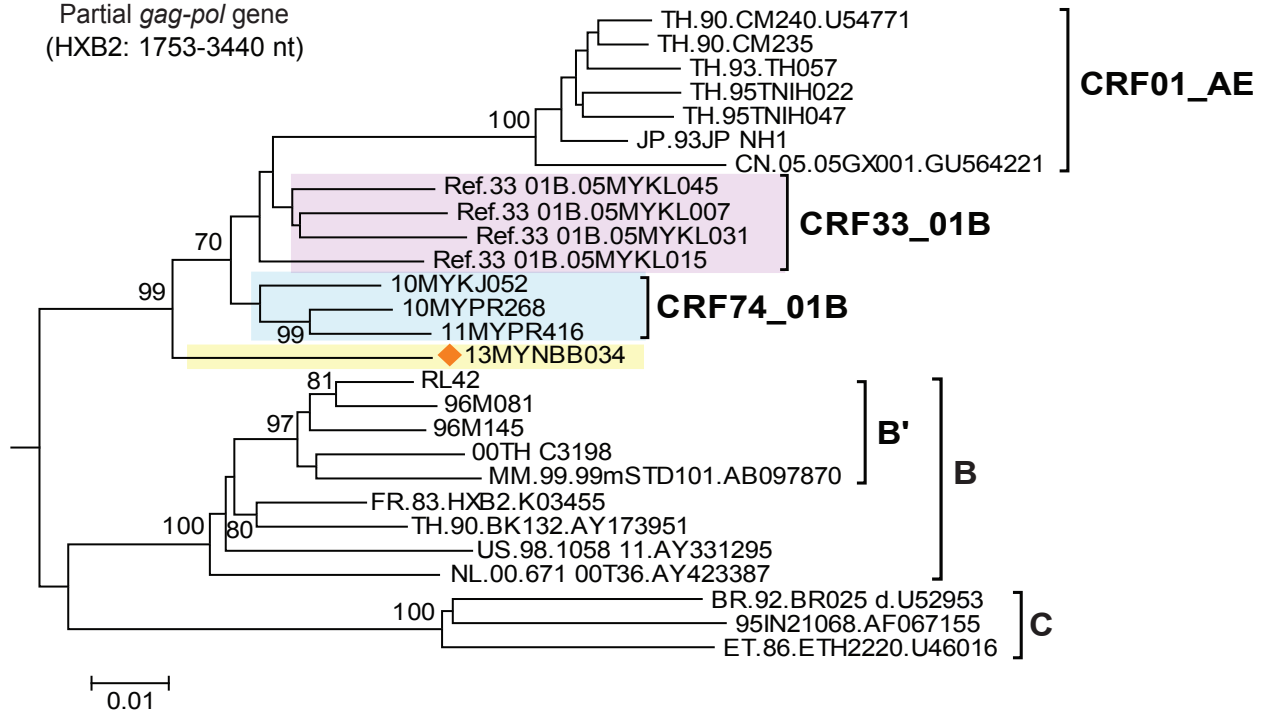

Supplement: S1 Fig — Phylogenetic tree was constructed in MEGA 5.05 using Kimura 2-parameter method of nucleotide substitutions and the reliability of the branching nodes were assessed by bootstrap analysis of 1000 replicates. The reference sequences were labelled in the following order: country of origin, year, isolate name and GenBank accession number. Abbreviations were used to indicate the country of origin. Bootstrap values of greater than 70% were indicated on the branch nodes. The scale bar represents 1% genetic distance (0.01 substitutions per site). (PDF) [file pone.0161853.s001.pdf]

Partial *gag-pol* gene  
(HXB2: 1753-3440 nt)

▲ Incident infection  
● Prevalent infection

Subtype G<sub>MY</sub>

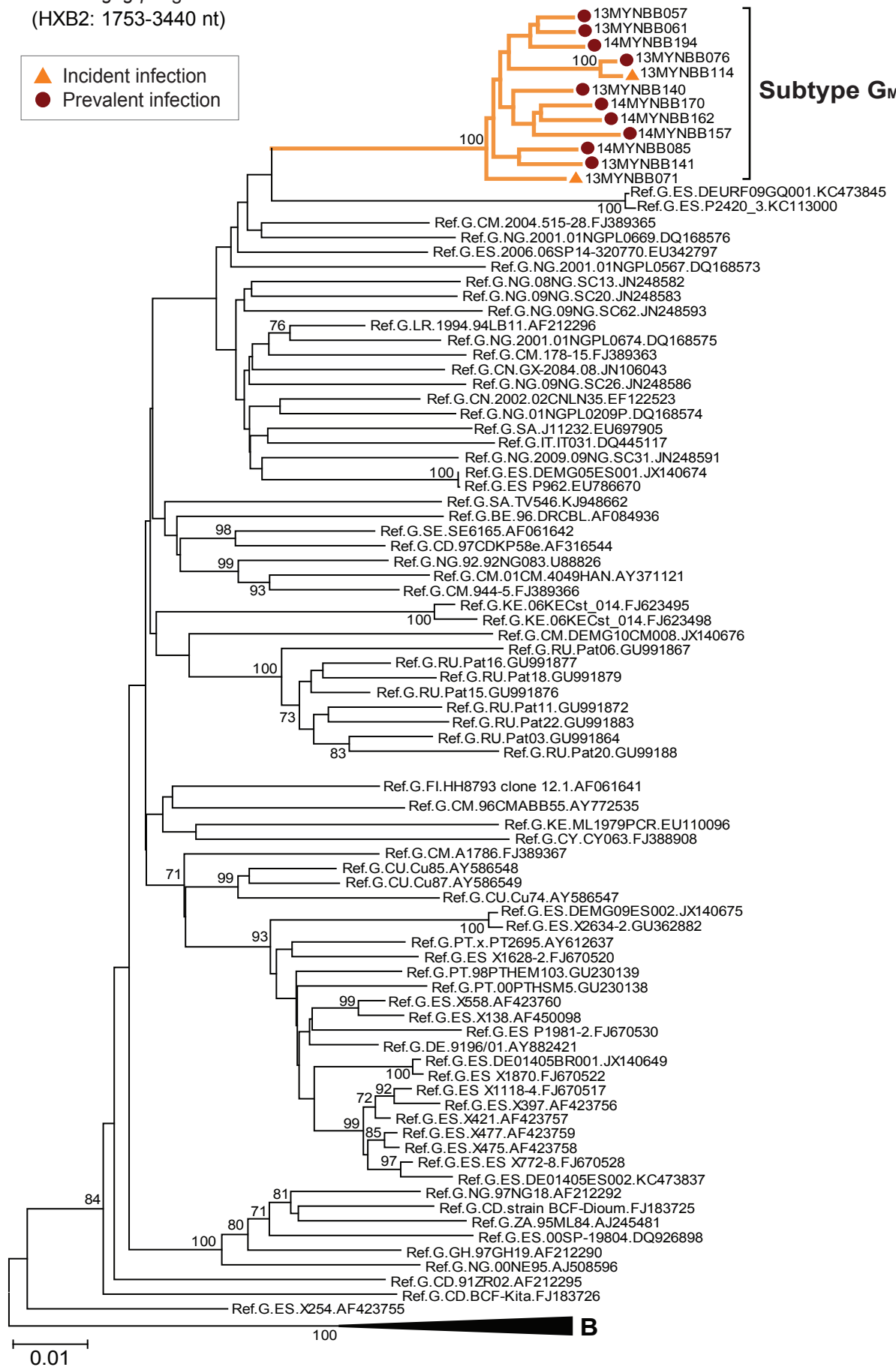

Supplement: S2 Fig — Neighbour-joining tree was constructed in MEGA 5.05 using Kimura 2-parameter method of nucleotide substitutions and the reliability of the branching nodes were assessed by bootstrap analysis of 1000 replicates. The reference sequences were labelled in the following order: genotype, country of origin, isolate name and GenBank accession number. Abbreviations used include MY, Malaysia and AF, Africa. Subtype B reference sequences were used as outgroup. Bootstrap values of greater than 70% were indicated on the branch nodes. The scale bar represents 1% genetic distance (0.01 substitutions per site). (PDF) [file pone.0161853.s002.pdf]
